# Integrated approaches for lameness control in free-stall dairy systems under heat stress and water scarcity

**DOI:** 10.1007/s11250-026-04948-4

**Published:** 2026-03-17

**Authors:** Nadia Hamdi Fahim

**Affiliations:** Animal Production Department, Faculty of Agriculture, Cairo University, Giza, Egypt

**Keywords:** Dairy cattle, Lameness, Heat stress, Water scarcity, Sensors, Machine learning

## Abstract

Lameness remains a critical welfare and productivity issue in dairy cattle, now intensified by climate-driven stressors such as heat stress and water scarcity. While each stressor has been widely studied individually, their combined and synergistic impacts on lameness risk are insufficiently characterized. This review synthesizes current evidence on the physiological, behavioral, and environmental pathways connecting these stressors to hoof health deterioration. It further inspects advances in sensor technologies and machine learning models. These models are capable of detecting and predicting lameness, heat stress, and water scarcity within integrated, multi-stressor monitoring frameworks. A comparative analysis was conducted for algorithms, including Random Forest, Gradient Boosted Decision Trees, Support Vector Machines, and deep learning-based pose assessment. The analysis revealed notable methodological and sensor overlap, allowing cross-application of validated models between domains. Such integration offers gains in cost-efficiency, data infrastructure use, and scalability, supporting the development of adaptable early-warning systems for precision livestock farming. However, regional climatic variability and infrastructure barriers still pose obstacles to adoption. There is a need for locally adapted thresholds, farmer training, and sustainable implementation strategies. By integrating climate-resilient housing, nutritional optimization, and AI-driven monitoring, dairy systems can transition toward anticipatory, resource-efficient management. This transition enhances welfare, alleviates lameness risk, and improves robustness under escalating environmental variability.

## Introduction

Lameness is one of the main causes of culling in dairy cows (Fahim et al. 2021). It is multifactorial, including infectious diseases, metabolic disorders, environmental, nutritional, and management factors. Lameness negatively affects animal welfare and productivity through the reduction of milk production and reproductive performance, and the increase of veterinary costs and culling rates (Tillack et al. 2024; Fahim et al. 2025a). It represented the third largest cause of economic loss in dairy units (Ozsvari 2017). The financial losses can exceed 40% based on the level of production (Urban-Chmiel et al. 2024). The cost was estimated to be between $116.8 and $264.7 per lameness case based on the severity (Sarıözkan and Küçükoflaz 2024).

Although the known effects of lameness have been well-reported, increasing interest has shifted to extrinsic factors that increase its prevalence and severity, especially in the face of the rising impacts of climate change. Among them, heat stress and water scarcity have become the crucial environmental variables that influence the health and the hoof of dairy cows. High ambient temperatures interfere with physiological homeostasis, elevated metabolic imbalances and body temperatures, and decreased feed and water intake (Beale et al. 2018).

Furthermore, high temperatures lead cows to behavioral changes such as increased standing for long periods to dissipate excess heat. This negatively affects hoof health, which may end with lameness (Habiba et al. 2025). Simultaneously, an insufficient water supply impairs hydration, immune response, and housing hygiene, which provides favorable conditions to infectious hoof diseases.

Despite well-documented impacts of heat stress and water scarcity, most research has studied them separately, leaving a gap in understanding their combined effects on lameness occurrence. Therefore, this review aims to address that gap. It explores current knowledge on how heat and water stress interact physiologically, behaviorally, and environmentally to increase lameness risk. It also investigates the potential of recent sensor technologies and AI-based machine learning in early detection of lameness, and prediction of heat stress and water scarcity. Additionally, it synthesizes integrated management approaches, including nutrition interventions, housing layout, and heat mitigation strategies. Overall, this work provides a practical framework for enhancing cow welfare, productivity, and farm sustainability under changing climatic conditions.

### Leading causes of lameness in dairy cattle

Lameness is strongly associated with the environmental, nutritional, and management conditions. Determining causes of lameness is essential to designing approaches that can diminish its risk. Digital dermatitis is one of the common causes of lameness. It is an infectious disease that is primarily characterized by inadequate hygiene conditions that can encourage the development of pathogenic organisms (Moreira et al. 2018; Urban-Chmiel et al. 2024). Another cause of lameness is sole ulcers and white line disease. These are mechanical diseases that are attributed to overworking of the hooves and improper hoof management, which causes damage to claw horn structures (Bell et al. 2022). Interdigital phlegmon is another infectious etiology of lameness that is closely related to poor environmental conditions. It can severely impair movement and lead to a great deterioration of welfare because of its rapid development (Blowey et al. 2007). Finally, laminitis is a complicated metabolic condition that may predispose animals to different hoof lesions. It is usually caused by metabolic disorders or long-term standing, which increase mechanical stress on the structures of the claws (Urban-Chmiel et al. 2024).

These issues are worsened with increased animal density in a given space, imbalanced nutrition, unhygienic barns, and exposure to harsh environmental conditions such as heat and dryness.

### Combined effects of heat stress and water scarcity on lameness in dairy cows

Climate-driven stressors, most notably heat stress and water scarcity, are associated with an increased lameness risk. However, the accumulating evidence is that they do not affect the health of the hooves only cumulatively. Instead, these stressors interact through interconnected physiological and biomechanical mechanisms and greatly increase the risks of lameness. These interactions indicate the existing limitations of single-stressor models. They also highlight the need to have a comprehensive explanation of how the combined effect of heat and water stressors has a synergistic, debilitating impact on the integrity of the hooves and locomotor functioning.

### Physiological pathways linking heat stress and water scarcity to hoof integrity

Physiologically, heat stress initiates several thermoregulatory responses to maintain the core body temperature. The redistribution of the blood to the vital organs and peripheral vasodilation helps in cooling the body. However, this reduces blood circulation to the more distant tissues, including the corium and claw horn (Cartwright et al. 2023; Garvey 2022). This reduces the circulation of blood that supplies oxygen and nutrients to continue the process of keratinization and horn regeneration, thereby lowering the quality of the horn of the claw and weakening the shock-absorbing properties of the digital cushion. At the same time, heat stress alters the metabolic priorities. It decreases the intake of dry matter, indirectly lowering the access to the nutrients that help maintain the hoof tissue on a long-term basis (Nzeyimana et al. 2023).

A lack of water aggravates these physiologic disturbances through different pathways. Water deprivation interferes with the state of hydration, electrolyte balance, and peripheral circulation, which are all essential in keeping the tissues in an elastic and metabolically stable status (Golher et al. 2021; Jovan and Milan 2025). The dehydration also restricts the blood circulation to the corium and the integrity of the horn and the soft tissue structures, which decreases the capacity to maintain the mechanical loading. Moreover, the deficiency of water adversely affects immune competence, and it puts cows at increased risk of having infectious hoof diseases, in particular, where hygiene is not at its peak (Garvey 2022; Burkhardt et al. 2024). The availability of water beyond its direct physiological effects has a profound impact on hygiene and cleaning efficiency in the barns. Decreased consumption due to the lack of water results in compromised cleanliness of the flooring and the proliferation of the pathogens of digital dermatitis and interdigital infection (Krauss et al. 2016; Le Riche et al. 2017).

### Biomechanical consequences of climate-induced physiological strain

The physiological loads from the heat stress and water deprivation are translated into great biomechanical issues in the hooves. Prolonged standing is a common thermoregulatory behavior exhibited by cows to facilitate heat dissipation (Cook and Nordlund 2009; Westin et al. 2016). Standing on the hind legs has been reported to reduce lying time, thereby delaying recovery of hooves already compromised by inadequate perfusion and dehydration.

As soon as the quality of the horn and integrity of the digital cushion are reduced, the ability of the hoof to absorb mechanical shock is reduced, further accelerating the development of lesions, including sole ulcers and white line disease (Urban-Chmiel et al. 2024). It is worth noting that these biomechanical stressors accumulate over time to result in progressive locomotor impairment rather than temporary impairment.

### Synergistic effects and feedback loops in lameness development

Notably, the two stressors: heat stress and water scarcity, exert a synergistic effect that is more destructive than the effect of both stressors taken singly. The loss of water through evaporation rises with the temperature, and hydration requirements get higher at a time when water availability may be low, and physiological demands increase (Golher et al. 2021).

Less tissue perfusion and derangements of metabolism, along with weakening due to dehydration, with augmented mechanical loading, compose a positive-feedback mechanism that strengthens itself. Weakened hoof functions favor pain and distorted gait within this cycle that causes a change in weight distribution and the occurrence of lesions (Cook and Nordlund 2009; Bellis et al. 2024). Fragility in climatic stress conditions, thus, must be considered as an emergent phenomenon because of co-existing environmental stressors and not a direct impact of individual etiological factors.

### Implications for lameness prevention and integrated management

**Fig. 1:**
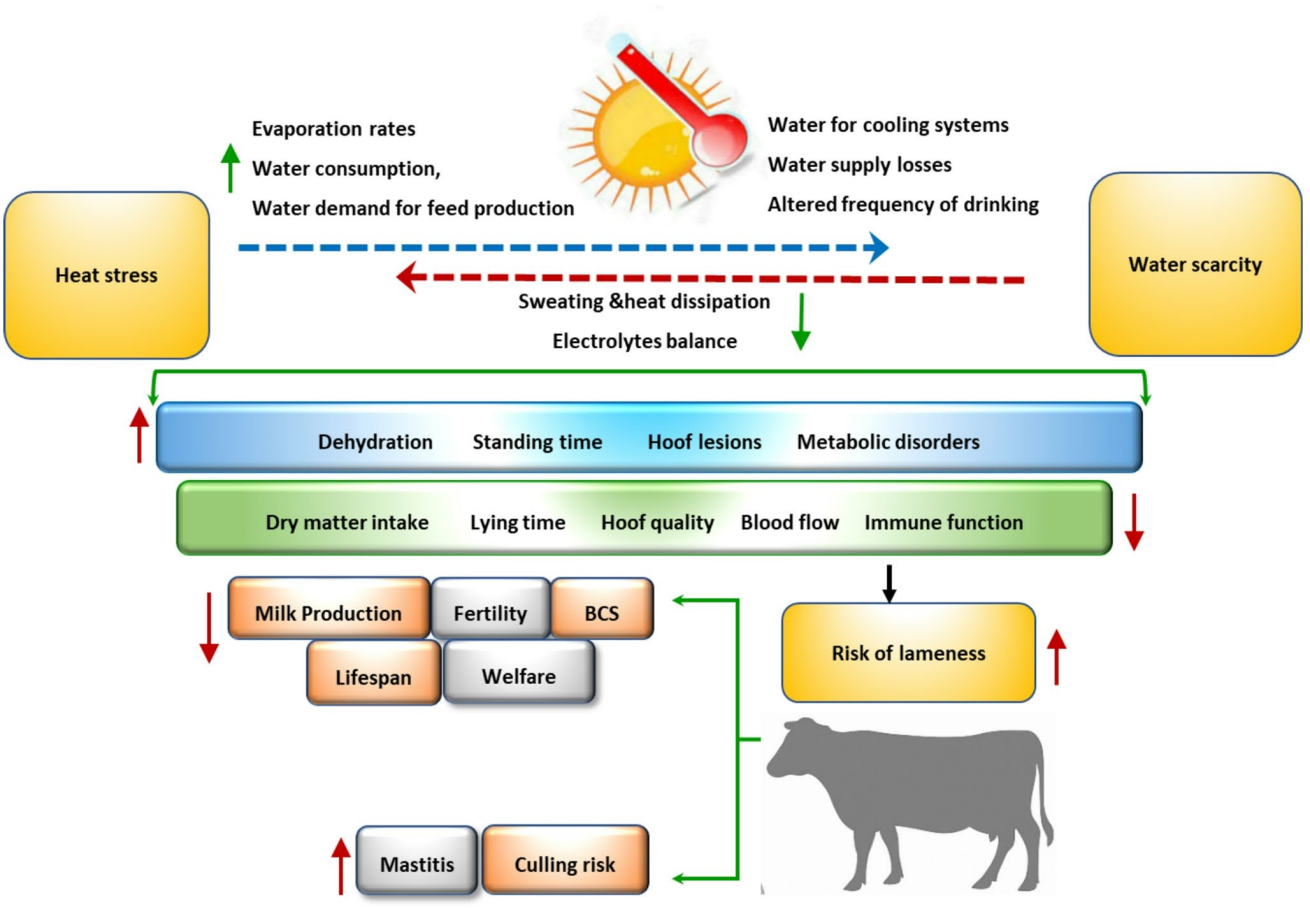
Interactive pathways linking heat stress and water scarcity to lameness risk in dairy cattle

This integrated perspective indicates a major flaw of the majority of current lameness prevention interventions, as they may consider heat stress, water management, or hoof care independently. Such forms of compartmentalized treatments are not able to explain the cumulative and interactive nature of climate-related stressors. The conceptual framework depicted in Fig. 1 shows that there is an inter-relationship between physiological strain, biomechanical overload, and progressive hoof pathology with environmental heat load and availability of water (Westin et al. 2016; Habiba et al. 2025; Fahim et al. 2025b). The understanding of these interdependencies is significant to the formulation of management, observation, and forecasting actions that can be used in mitigating the danger of lameness with increasing climatic disparity.

### Management strategies for lameness prevention under heat stress and water scarcity

Standard dairy farm management depends mainly on preventive procedures to keep the productivity and sustainability of animals. This part aims to combine traditional preventive care practices with emerging adaptive measures to address the mounting difficulties of climate change. The focus is on keeping hoof health in dairy cattle as well as mitigating the impacts of heat stress and drought.

### Housing design and bedding management

Housing design plays a great role in keeping the hoof health of dairy cows, especially under high temperatures and insufficient water conditions. The type and condition of floors are necessary; cushioning and non-slipping floors help in minimizing mechanical trauma and falls (French et al. 2023). Comfortable and non-slip floors ensure that the cows do not spend a long time standing on the wet floor, which is a significant predisposing factor for hoof diseases, and the water flows effectively. This reduces the amount of moisture in the environment (Garvey 2022). Barns and holding pens where the artificial or natural shade is included result in a lower heat load. Body temperature of animals can also be decreased further by the use of fans, misters, or evaporative cooling systems (Fournel et al. 2017; Liu et al. 2024). The positioning of such systems strategically around water points encourages frequent drinking, thermoregulation, and elimination of unnecessary spillage of water. Proper drainage systems also ensure hygiene, avoid the growth of bacteria, and allow cleaning and cooling water to be recycled to save resources (Skrydstrup et al. 2020).

The farm layout must also be in such a way that the walking distances between the housing, feeding, and milking areas are minimized. The impact of excessive walking up to 4 km/day has been linked to a greater duration of grazing, less time ruminating, fatigue of legs, and less time lying, contributing to the risk of lameness (Neave et al. 2021). Optimization of distances enhances the comfort of locomotion and promotes the health of the hooves.

Best bedding management is at the center of ensuring the integrity of the hoofs and the comfort of the cows. To evenly distribute the body weight, reduce the load and pressure of the hooves and joints, and decrease the occurrence of hoof lesions, cubicles should have adequate space and comfortable bedding material, such as sand or sawdust (Norring et al. 2008; Adams et al. 2017). It is essential to keep the bedding dry and clean because moisture powerfully exposes an individual to the chances of contracting infectious foot diseases, such as digital dermatitis (Sölzer et al. 2025).

### Water availability and management

Sufficient water supply and quality are two fundamental factors of thermoregulation, hoof health, and welfare. Moreover, the water points should be in proximity to the feeding and milking areas to reduce the competition, queuing time, and standing time (Monteiro et al. 2023). The continuous availability of cold and clean water contributes to cooling in case of a heat load (Reddy et al. 2023). Furthermore, maintaining water Hygiene ensures the regularity of drinking and discourages cows from seeking contaminated sources. The application of water supply based on the ambient temperature or milk production assures the hydration and helps reduce the risk of lameness (Giri et al. 2020).

Moreover, occupancy-controlled sprinklers and float-controlled or demand-activated water dispensers are some of the automated systems that improve the efficiency of water use and avoid unnecessary spillage (Monteiro et al. 2023). Parlor cleaning wastewater can be recycled and used in cleaning barns or to irrigate crops, which saves freshwater and protects hygiene and reduces the occurrence of hoof disease (Skrydstrup et al. 2020).

Overall, combining effective water management with the design of housing and bedding creates an environment that reduces the heat load. This keeps the body hydrated and helps preserve the integrity of the hooves. Hydration minimizes lameness and improves the welfare of dairy cows in the environment with climate-related stresses.

### Feeding management and interventions

Nutritional management is critical towards alleviating the incidence of lameness in dairy cows, especially in the face of escalating heat and water stress levels, which accompany climate change. Preventive feeding plans contribute greatly to the minimization of metabolic disorders, with predisposing effects on cows to the development of hoof pathologies (Langova et al. 2020).

Consumption of high quantities of rapidly fermentable carbohydrates, such as most diets high in grain, with a disproportionate ratio of non-fermentable carbohydrates to structural fiber, will alter the dynamics of ruminal fermentation. Such dysregulation supports lactic acid synthesis. It also encourages the discharge of vasoactive mediators, such as histamines and endotoxins. This disrupts microcirculation in the hooves, resulting in laminitis and consequently lameness (Lean et al. 2013). Meanwhile, the regulation of dietary fiber and fat levels reduces the amount of heat producedby the metabolism (Conte et al. 2018).

Ensuring that the diet is consistent through the use of total mixed rations would reduce the sudden changes in the nutrition levels. Such changes can cause metabolic instability and predispose cows to develop lameness. At the same time, the optimization of feed intake would ensure the balance of water and indirectly maintain the condition of the hoof (Nzeyimana et al. 2023). Besides, balanced rations increase the efficiency of the feeds and minimize the unnecessary water consumption due to the inability to utilize the nutrients.

In addition, trace minerals, including copper, zinc, and manganese, as well as biotin, were found to be consistently associated with increased claw horn strength and a decrease in the prevalence of lameness. Since the positive changes in the health of claws demonstrated through the use of monensin supplementation are not conclusive yet, the first results indicate that there might be some positive consequences (Langova et al. 2020). Furthermore, antioxidants and modified proteins had a beneficial effect on thermotolerance and immune response. They are incorporated into the diet, providing additional resistance to heat-induced metabolic stress and its subsequent impact on the state of the hooves (Peretti et al. 2022; Chen et al. 2023).

Furthermore, proper body condition also adds to the mechanical strength of the digital cushion in terms of increasing the shock absorption ability and decreasing the occurrence of both sole ulcers and white line disease (Kranepuhl et al. 2021; Wilson et al. 2021).

Generally, in a hot environment, planning the timing of feeding during cooler periods can be an effective method to reduce the amount of heat generated in metabolic processes related to digestion, and in turn reduce the total thermal load.

### Trimming the hooves regularly

Frequent trimming is an important prevention method. It helps to prevent overgrowth of the hooves, reduce digital dermatitis and sole ulcers, two major causes of lameness (Stoddard et al. 2025). It has been demonstrated that efficient trimming techniques redistribute weight more equally and increase frictional qualities at the contact of the floor and the claw. This maintains the size of hooves in an optimal size, allowing animals to move as usual (Dahlvik et al. 2024). Moreover, regularly trimmed cows have a reduced growth rate of hooves. This reduces the rate of lameness of indoor and outdoor systems (Daros et al. 2022). In addition, Waldbauer et al. (2024) showed that radical trimming regimens- trimming 50% of the herd after every bi-monthly- were found to be more economical than blanket treatment plans under the studied conditions, with the total savings in three years being more than 4000 dollars.

### Routine footbath protocols

Footbaths constitute an essential component of hoof care regimes. Footbaths with copper sulfate or zinc sulfate can greatly decrease hoof infections (Prastiwi et al. 2019). Twice-weekly application of an iron, zinc, and aluminum-based hoof spray reduced the risk of active digital dermatitis lesions by 4–6 fold. (Grimm et al. 2025). Daily monitoring of the cows and early action among the lame cows minimizes economic loss as well as improves animal welfare (Thomsen et al. 2023).

### Education of farmers and stakeholders’ engagement

Lameness is multifactorial, which complicates its control due to human behavioral factors (Pedersen 2025). Therefore, lameness control success is based on education and active cooperation of all stakeholders involved in the dairy farming system (Roche et al. 2024).

For example, the COM-B model, developed by Clark et al. (2024), introduced three determinants of the behavior change of farmers: capability, opportunity, and motivation. They found that the improved communication between farm advisers and farmers enhanced treatment compliance and reduced the incidence of lameness.

In addition, the DairyCo Healthy Feet Program is one of the many education schemes where farmers and veterinarians receive extensive training (Atkinson 2013).

Collectively, these management interventions should be implemented in a coordinated manner; applying some while ignoring others, particularly in conditions of heat stress and water scarcity, will not achieve the desired outcomes of preventing lameness in dairy cattle.

### Sensor-machine learning approaches for managing lameness under heat stress and water scarcity

Early lameness detection enables the burden of early and precise management interventions. The earlier lameness is detected, the higher the chances of effectively minimizing its negative impacts (Af Sandeberg et al. 2023; Siachos et al. 2024).

Sensor technologies and machine learning (ML) models have become an integral component of the dairy management system. They are capable of simultaneously monitoring, controlling, and predicting complex welfare outcomes. Such a combination can be considered the transition to proactive, data-driven management in the context of contemporary dairy production systems, as opposed to descriptive assessment of welfare. Thus, sensor technologies and ML models could be holistically integrated to diminish lameness in the expanded framework of climatic stress.

### Sensor technologies as multidimensional stress tools

The sensor technologies define the basis of the observational layer of the lameness monitoring system (Table 1). Simultaneously, sensors record behavioral and physiological indicators of heat stress and water accessibility. Gait dynamics, activity patterns, and lying-standing behavior are directly related to locomotor health. They are monitored by wearable accelerometers on the leg, neck, or ear and are sensitive to heat stress and water availability (Jarchi et al. 2021).

**Table 1:** Sensor-based approaches for early lameness detection in dairy cattle

Sensor / Method	Description	Key capabilities	Notes	References
Leg-worn accelerometers	Measure 3D motion and gait parameters	Acceleration, stance time, asymmetry	Most common wearables for direct gait metrics	(Pastell et al. 2009; Haladjian et al. 2018; Jarchi et al. 2021)
Neck/ear accelerometers	Monitor activity and posture changes	Lying/standing bouts, rumination, restlessness	Early behavioral anomaly detection	(Poursaberi et al. 2011)
Computer vision systems	Video-based locomotion analysis	Hoof trajectory, joint motion	Enables hands-free automated gait scoring	(Kang et al. 2020; Myint et al. 2024)
Combined multi-sensor systems	Fusion accelerometers, video, and force data	Temporal-spatial gait integration	Improves detection accuracy via data synergy	(Dhaliwal et al. 2024)
Kinetic methods	Pressure-sensitive walkways and force plates	Detect asymmetric weight bearing	Biomechanical gait analysis complement	(Alsaaod et al. 2019; Bellis et al. 2024)
Metabolomics profiling (LC-MS)	Biomarker identification via metabolic signatures	Molecular indicators of lameness	Emerging complementary approach	(Randall et al. 2023)
Internet of things (IoT) and big data analytics	Networked sensor data and advanced analytics	Scalable automated data collection and processing	Integrates into precision livestock systems (PLF) for farm-wide decision support	(Qiao et al. 2021; Qazi et al. 2024)

Computer vision has furthered this ability by allowing automated and contact-free gait analysis and posture analysis to enable continuous monitoring in commercial farm management without increasing animal handling (Myint et al. 2024). These systems are valuable in large herds, where visual scoring cannot be conveniently used, and the environment can conceal the expression of overt lameness.

Multi-sensor systems that integrate accelerometers, video information, and kinetic data offer a more robust and context-sensitive description of animal reactions in comparison to single-sensor frameworks (Dhaliwal et al. 2024). When cows are exposed to heat stress or water deprivation, they tend to respond with compensatory behaviors such as more standing time or redistribution of weight, before clinical lameness is evident. Kinetic devices, such as force plates and pressure-sensitive walkways, monitor early signs of a biological overload and early signs of a developing hoof pathology by assessing asymmetric loading of limbs (Alsaaod et al. 2019; Bellis et al. 2024).

In addition to biomechanical sensing, Table 1 shows a biochemical component to lameness monitoring based on metabolomics profiling. Molecular signatures of lameness have been determined with the help of ML-assisted metabolomics. Though not yet implemented on a large-scale farm basis, the omics-based indicators enable useful physiological validation as well as shed light on systemic stress reactions that do not yet result in overt locomotor impairment, especially in the case of long-lasting heat and water stress (Randall et al. 2023).

### Machine learning models for lameness as a core analytical framework

Based on these diverse sensor measurements, the ML models presented in Table 2 provide the analytical core of early lameness detection and present transferable frameworks of integrated stress measurements. Classical algorithms such as AdaBoost and Support Vector Machines (SVMs) are also applicable to image and video-based gait classification and provide stable baseline performance under controlled laboratory conditions (Myint et al. 2024). Nevertheless, they are susceptible to external sound, which reduces their robustness in cases where heat stress or dehydration causes deviation in normal behavioral expression.

**Table 2:** Machine learning-driven sensor technologies for early lameness detection in dairy cattle

Model/Approach	Sensor/Data input type	Key detection features	Performance metrics and highlights	Applications and notes	References
AdaBoost	Image/video gait and posture (Computer Vision)	Hoof placement, stride tracking, abnormal gait	Accuracy: 77.9%. Robust image processing with deep learning	High-performance visual gait analysis	(Myint et al. 2024)
Support Vector Machine (SVM)	Image/video gait features	Posture, stride features	Accuracy ~ 75%. Classical model for video-based gait analysis	Reliable baseline for visual detection	(Myint et al. 2024)
Random Forest (RF)	Accelerometers (leg, neck, ear)	Lying time, activity, step count	Accuracy > 90% with the ROCKET classifier. Strong predictive power	Sensor-based time-series classification	(Neupane et al. 2024)
Gradient Boosted Decision Trees (XGBoost, GBDT)	Multisource: accelerometer + milk yield + live weight	Behavioral + production data fusion	AUC 85%; Sensitivity/Specificity ~ 78%. Commercial farm use	Data fusion improves detection precision	(Borghart et al. 2021)
Deep Learning (CNNs, Pose Estimation)	Multimodal computer vision (gait + facial)	Joint angles, movement symmetry, and facial tension	Highest accuracy; scalable real-time detection	State-of-the-art, automated herd monitoring	(Dhaliwal et al. 2024; Li et al. 2024)
Naïve Bayes	Various (video, sensor data)	Probabilistic feature modeling	F1-score superior to logistic regression in some datasets	Simpler probabilistic baseline or ensemble member	(Shahinfar et al. 2021)

Sensor-based models, especially the Random Forest (RF) classifiers, are applied to accelerometer time-series data. Particularly, these models are useful in describing the slight, early deviations of activity and mobility that develop as a result of the joint action of heat, water, and hoof stress (Neupane et al. 2024).

Others, such as gradient-boosted decision trees (GBDT/XGBoost), are more sensitive and specific (balanced), as they combine behavioral sensor data with production variables, which are milk yield and live weight, thereby making them suitable in on-farm applications (Borghart et al. 2021). The integration of behavioral and production data is valuable, particularly during climate stress. During stress, the milk yield starts to decline before the abnormalities in the gait, meaning that such models can be used as proactive control methods, rather than reactionary monitors.

Consequently, deep learning models such as convolutional neural networks and pose estimation models are the most sophisticated level of analysis. These models reach generalized patterns of distress compared to stressor-specific cues. They are, therefore, inherently in good positions to predict various stressors, which is accomplished via learning common representations of gait asymmetry, posture, and facial tension (Dhaliwal et al. 2024; Li et al. 2024). They are scalable and can be automated with the possibility of amplifying continuous monitoring of herds at the herd level under varying conditions.

Naïve Bayes classifiers are also complementary ML models, which are used in integrated pipelines. Despite being computationally easy, these probabilistic models have been shown to have higher F1-scores than logistic regression on certain datasets. They are suitable as a baseline classifier or an ensemble part, especially in resource-constrained systems (Shahinfar et al. 2021).

### Heat stress monitoring as a preventive control strategy for lameness

Table 3 indicates that one of the required upstream factors of lameness control is heat stress monitoring systems. The thermal load is commonly measured using environmental sensors that are able to measure the temperature, humidity, wind velocity, and solar radiation. However, physiological indicators such as the temperature of the skin, respiratory rate, and rumen temperature should be prioritized as they are more accurate in expressing the thermal load that happens, and then the locomotor consequences occur (Huang et al. 2023; Mylostyvyi et al. 2024).

**Table 3:** Machine learning-driven sensor technologies for monitoring heat stress in dairy cattle systems

Model	Data input	Sensors	Performance	Application	Reference
Random Forest	Environmental + physiological data	environmental, skin temperature, respiration	Good predictive performance	Real-time alerts; triggers cooling (shade, sprinklers)	(Georgiades et al. 2025)
Gradient Boosted Machines (XGBoost)	Environmental + physiological data	environmental, skin temperature, respiration	AUC ~ 0.85; sensitivity and specificity ~ 78%	Early warning system for heat stress severity	(Becker et al. 2021)
Artificial Neural Networks	Time-series environmental + physiological data	rumen temperature bolus, respiration, environmental	High accuracy in core body temperature prediction	Forecasting for proactive heat stress management	(Chapman et al. 2023)
Cubist Regression Model	Rumen temperature + weather data	rumen bolus, environmental	Sensitivity 79%, precision 52%	Early alerts for timely heat abatement deployment	(Woodward et al. 2024)
Logistic Regression	THI, respiration rate, behavior, milk production traits	environmental, respiration, accelerometer	Effective in moderate heat stress detection	Evaluation of cooling system effectiveness	(Becker et al. 2021)
Naïve Bayes	Environmental + physiological data	environmental, respiration, accelerometer	Effective in moderate heat stress detection	Aids early intervention strategies	(Becker et al. 2021)
Support Vector Machines	Thermographic, physiological, and environmental data	infrared thermography, environmental	Accuracy up to 86.8%	Non-invasive, rapid heat stress classification	(Pereira et al. 2024)

The gathered data are sent to models such as GBDT, neural networks, Cubist regression, and probabilistic classifiers to generate early warning signals and take timely cooling responses (Chapman et al. 2023; Woodward et al. 2024).

Heat stress mitigation indirectly lowers dehydration, standing times, and peripheral circulatory load, which are biomechanical and inflammatory antecedents of lameness. In this regard, the thermal monitoring is not an analogous welfare measure, but, conversely, a preventive control pathway, which was incorporated into the lameness management (Becker et al. 2021).

### The water availability monitoring and behavioral inference

Another necessary level of integrated welfare control is sensor technologies to monitor water access, which are described in Table 4. RFID-tagged troughs, intelligent drinkers, flow meters, and level sensors will deliver real-time measurements of drinking habits and water supply, which will allow supply disruptions or lower consumption to be detected in advance (Tang et al. 2021).

**Table 4:** Sensor technologies for monitoring water access in dairy cattle systems

Sensor /Technology	Measured /Parameter		Key data /Signals	Role in detecting water scarcity	Examples/Notes	References
RFID-tagged troughs and smart drinkers	Individual drinking behavior (volume, time)		Time-stamped drinking events per cow	Detects abnormal drinking patterns linked to water scarcity or welfare issues	RFID-integrated troughs; automated drinkers	(Tang et al. 2021)
Flow meters and level sensors	Usage and trough/tank availability		Flow rate, level trends	Identify supply disruptions or trough depletion	Smart meters, reservoir level monitors	(Tang et al. 2021)

The low consumption of water is closely related to behavioral changes like an increased standing period and decreased lying (O’Connor et al. 2022). These behaviors all increase the risk of lameness. Activity data obtained by accelerometers are thus proxy measures of water stress, which supports the interrelation between these systems of monitoring (De Kok 2024).

### Machine learning models for predicting water scarcity

As displayed in Table 5, numerous algorithms that have been used to detect lameness can also be used to detect water shortage by using indirect behavioral and physiological cues. RF, SVMs, ANN, GBDT, and deep learning-based vision models would be able to use accelerator data, gait features, and production metrics to infer deviations in the hydration status and water intake behavior (Shine et al. 2018; Osaki et al. 2024).

**Table 5:** Machine learning-driven sensor technologies for monitoring water scarcity in dairy cattle systems

Model	Sensor/Data type	Key features and Performance	Applications	References
AdaBoost	Image/video (gait, posture)	Accuracy: 77.9%; detects hoof placement, stride, abnormal gait	Robust computer vision for gait analysis	(Myint et al. 2024)
Support Vector Machine	Image/video gait features	Accuracy ~ 75%; posture and stride detection	Classical baseline for video analysis	(Myint et al. 2024)
Random Forest	Accelerometers (leg, neck, ear)	Accuracy > 90%; step count, activity, lying time	Time-series sensor classification	(Neupane et al. 2024)
Gradient Boosted Decision Trees (XGBoost/GBDT)	Multisource: accelerometers + milk yield + live weight	AUC ~ 85%, sensitivity and specificity. ~78%; behavior + production fusion	Enhances detection via data integration	(Borghart et al. 2021)
Deep Learning (CNNs, Pose Estimation)	Multimodal computer vision (gait + facial)	Highest accuracy; analyzes joint angles, symmetry, and facial tension	Real-time, scalable herd monitoring	(Dhaliwal et al. 2024; Li et al. 2024)
Naïve Bayes	Mixed: video and sensor data	Outperforms logistic regression in some datasets; probabilistic modeling	Lightweight model or ensemble component	(Shahinfar et al. 2021)

It is important to note that predictive accuracy can also be achieved even more by combining the outputs of accelerometers with the information provided by automatic milking systems (Lemmens et al. 2023). This example is indicative of how streams of production and behavioral data can be re-utilized in other stress areas without the need to invest in more sensors.

### Combined interpretation and conceptual integration

**Fig. 2:**
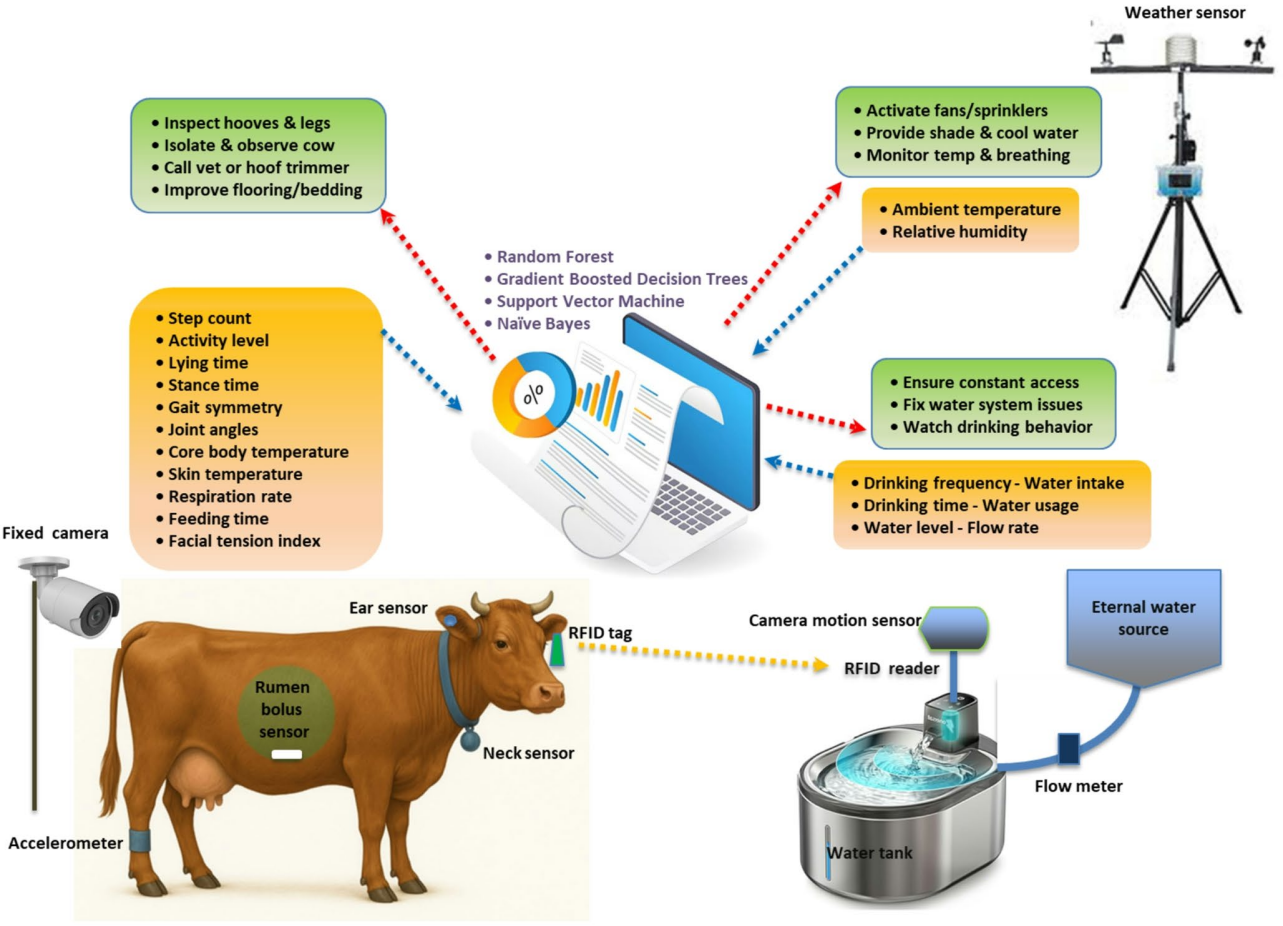
Integrated machine learning models for managing lameness, heat stress, and water scarcity in dairy cattle

The schematic architecture of the integrated machine-learning-based control approach is shown in Fig. 2. It links sensors and analytics with the management interventions in the lameness, heat stress, and water scarcity domains. The framework can be described as compromising four interrelated layers: (1) a sensing layer that captures behavioral, physiological, biochemical, and environmental signals; (2) a data fusion and preprocessing layer that synchronizes multimodal signals; (3) an ML analytics layer, which relies on those models, which are summarized in Tables 2 and 3, and 5 to produce animal-level risk probabilities; and (4) a control and decision-support layer to trigger adaptive interventions, including targeted cooling.

All of these layers make it possible to implement a predictive-preventive paradigm whereby initial sensor-based identification guides an adaptive decision-making process by ML, and the loop between monitoring and control is bridged. It is in this sense that the architecture in Fig. 2 captures the shift in the descriptive surveillance towards intelligent and self-regulating welfare control in dairy systems under the growing climatic uncertainty.

### Considerations and regional limitations in adopting the heat stress–water scarcity models

The issue of regional climatic heterogeneity is one of the still significant limitations to global implementation of ML-based models to manage heat stress and water shortage in dairy production systems. Despite the considerable potential of these models in enhancing the process of decision-making as well as animal welfare, the performance, scalability, and transferability of these models have been highly affected by regional climate, infrastructure, and management practices.

The majority of more advanced ML systems combine heat factors of the environment, such as ambient temperature, relative humidity, and solar radiation. Physiological factors, such as core body temperature and respiration rate, have been trained and tested in the regions of Europe and North America, where temperatures are relatively low. In such settings, penalty regression models and gradient boosting machines have proven to be effective in predicting the occurrence of heat stress, which can be used to take appropriate measures to mitigate it (Becker et al. 2021). Similarly, localized methods like adaptive regression trees have also been integrated in heat-health early warning systems in countries such as France and the United Kingdom to protect animal life during extreme heat events (Masselot et al. 2021).

Nevertheless, the success of these methods cannot be generalized. The differences in the efficiency of evaporative cooling also restrict the usefulness of the interventions to be applied to arid or subtropical conditions in the humid tropical areas, where physiological cooling capacity is limited (Fournel et al. 2017). On top of the climatic variability, high costs of implementation, technical complexity, and poor digital infrastructure are further barriers to adoption in the developing regions. This means that local validation and calibration must be rigorous, and algorithmic thresholds, sensor network architectures, and implementation strategies would need to be validated for region-specific patterns of heat stress and water availability. To a large extent, these challenges are contextual and infrastructural and not necessarily weaknesses in the modeling approaches themselves.

### Recommendations

The high-level ML-based structures cannot be modified into farm-based management tools as easily as a technological one due to the high level of regional variation in climate, infrastructure, and availability of resources. This can be successful if supported with proper training. The capacity to train and educate the farmers, veterinarians, and technical people on the model outputs should be supported by certain capacity-building activities so that they can be in a position to interpret and apply them as a component of the day-to-day management decisions. Participatory training and extension activities can be extremely helpful in helping bridge the gap between technological innovation and on-farm practice.

At the same time, the development of regionally optimized and low-cost sensor platforms that improve access and long-term sustainability, especially in limited-resource settings, is also necessary. The ongoing support of open-access data ecosystems will also facilitate the localization of the calibration, knowledge exchange, and enhance the usability of the models in the various production systems. Policy interventions are also important, and financial obstacles should be reduced through incentives, subsidies, and joint implementation schemes, or even through promotion of animal-health technologies, and equal application should be supported. The concerted efforts could make precision livestock technologies scalable, responsive solutions for the farmers once they are combined into more comprehensive climate-resilient livestock development strategies.

### Future research directions

Although the sensor technologies that are currently available are built on the idea of tracking the behavioral, physiological, and biomechanical signs of lameness, heat stress, and water availability in high-resolution and continuous, their effectiveness during the extreme climate conditions has not been adequately tested yet. The outcomes of the accuracy decrease when operating in high thermal load, or the stress of water conditions substantiate the fact that the large-scale validation is needed when the actual farm conditions are considered, but not when the actual conditions are under controlled research conditions. The future research should then focus on designing multi-label datasets, establishing region-specific calibration protocols, and subjecting these procedures to extreme climatic conditions to validate the research.

The objective of research must be to develop complete cross-functional modeling systems that will independently model thermal load, conditions of hydration, and locomotor wellness on the same predictive models. The resilience and model transferability over time in dissimilar production systems should be tested using field-scale and longitudinal studies. Moreover, explainable artificial intelligence with mechanistic physiological modeling can also prove helpful in identifying causal mechanisms to describe stress-lameness relationships compared to using correlative predictions. It is also important to note that lightweight algorithms must be developed, and their financial feasibility and welfare considerations must be considered systematically in the data-limited scenario and implemented in real-time.

## Conclusion

This review highlights that it is ineffective to manage lameness in dairy cattle without considering the climate-induced stressors, such as heat stress and water scarcity. These factors have been demonstrated to interact at a physiological, behavioral, and environmental level to predispose animals to lameness. The sensor technologies and machine learning models that were initially developed to monitor a single stressor have converged into complex multi-stressor models, which are capable of simultaneously monitoring lameness, thermal load, and hydration status. This integration provides early-warning capabilities and optimization of resources and scalable decision support in various production environments.

However, the economic constraint, infrastructure barriers, and the fact that these methods demand rigorous regional calibration to address climatic heterogeneity present a challenge to the potential of these models. These barriers can be defeated by a concerted effort to invest in climate-resilient infrastructure, educate farmers, enable data access, and provide policy incentives to ease the adoption. By inserting sensor-machine learning systems within the broader dairy management strategies, including housing design, nutritional balancing, and particular cooling and hydration methods, the dairy operations can be transformed into precision-based, climate-resilient production systems. This paradigm shift will be important to safeguard animal welfare, sustain productivity, and build resilience in the face of escalating environmental variability.

## Data Availability

Not applicable.
